# Recent advances in understanding cardiac contractility in health and disease

**DOI:** 10.12688/f1000research.8661.1

**Published:** 2016-07-20

**Authors:** Ken T. MacLeod

**Affiliations:** 1 Faculty of Medicine, National Heart & Lung Institute, Imperial College London, London, UK

## Abstract

The aim of this review is to provide the reader with a synopsis of some of the emerging ideas and experimental findings in cardiac physiology and pathophysiology that were published in 2015. To provide context for the non-specialist, a brief summary of cardiac contraction and calcium (Ca) regulation in the heart in health and disease is provided. Thereafter, some recently published articles are introduced that indicate the current thinking on (1) the Ca regulatory pathways modulated by Ca/calmodulin-dependent protein kinase II, (2) the potential influences of nitrosylation by nitric oxide or S-nitrosated proteins, (3) newly observed effects of reactive oxygen species (ROS) on contraction and Ca regulation following myocardial infarction and a possible link with changes in mitochondrial Ca, and (4) the effects of some of these signaling pathways on late Na current and pro-arrhythmic afterdepolarizations as well as the effects of transverse tubule disturbances.

## Introduction

In the last year or so, which areas of cardiac physiology research have seen significant developments? In the following article, I have gathered a small selection of articles published last year (2015) that illustrate some emerging concepts, challenge existing dogma, and perhaps provide food for thought.

I am conscious that some people reading this article may not have specialist knowledge of the field, so, to help define various terms and provide some context for the slightly disparate collection of articles, I start with a short introduction to cardiac contraction in health and disease.

### Cardiac contraction in the normal heart

In the normal heart, the coupling of electrical excitation (the action potential) to the production of contraction (EC coupling) involves the interaction of a number of cellular proteins involved in calcium (Ca) homeostasis ^1^. Ca influx through mainly L-type Ca channels in the surface membrane promotes further release of stored Ca from the sarcoplasmic reticulum (SR) via the SR Ca-release channel (the ryanodine receptor, RyR) by a process known as Ca-induced Ca release ^2^. Both fluxes of Ca combine to initiate contraction.

**Figure 1.**
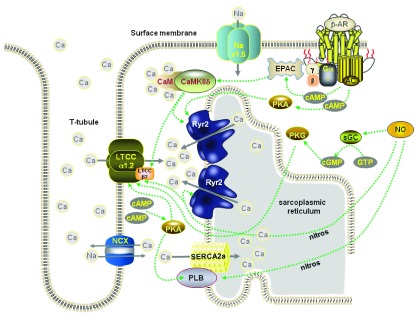
Schematic diagram of the main proteins involved in EC coupling in a ventricular myocyte and the main mechanisms for their phosphorylation or nitrosylation. LTCC = L-type Ca channel, Cav1.2; Nav1.5 = cardiac isoform of the Na channel; NCX = Na/Ca exchange; Ryr2 = ryanodine receptor; PLB = phospholamban; AC = adenylyl cyclase; sGC = soluble guanylyl cyclase; EPAC = exchange protein activated by cAMP; PKA = protein kinase A; PKG = protein kinase G.

The SR is a network of interconnecting tubules and cisternae that surround the myofibrils. At multiple sites within this network, the tubule membranes broaden to form flattened sacs, the junctional SR cisternae, which lie adjacent to the surface membrane, and its extensions, the transverse (T)-tubules. The surface membrane and T-tubules that face the junctional SR membrane contain L-type Ca channels, and embedded in the apposing areas of the junctional SR membrane and grouped in clusters are the RyRs ^3, 4^. There is very close spatial apposition between the Ca channels and the underlying clusters of RyRs and so the inner parts of the Ca channels and the cytosolic parts of the RyRs exist in a microdomain with restricted ionic diffusion. In normal ventricular muscle cells, the T-tubule network allows essentially simultaneous activation of the Ca channels located on the surface and in deeper regions of the cells. This promotes effective EC coupling and allows synchronous Ca release throughout the interior of the cell ^5^. The small (about 2–5 μm diameter) local Ca releases that occur within the microdomains can be observed using confocal imaging techniques and Ca-sensitive fluorescent indicators and are termed “Ca sparks” ^6, 7^. The sparks represent the building blocks of EC coupling because the increase in cytosolic Ca in the entire cell that initiates contraction is produced by the temporal summation of the individual Ca sparks (see Figure 1).

Two main systems are involved in removing Ca from the cytosol and so inducing relaxation. Ca is pumped back into the SR by the phospholamban (PLB)-regulated Ca ATPase (SERCA) and extruded from the cell by the sarcolemmal Na/Ca exchange (NCX) ^1^. Though there are species differences, SERCA and NCX contribute about 70% and 25%, respectively, towards relaxation ^8^. In steady-state conditions, the amount of Ca leaving the cell is the same as the amount entering so that precise Ca homeostasis is achieved ^9^. The phasic increase and decrease of Ca that gives rise to the elements of contraction and relaxation, respectively, is generally termed the “Ca transient”. Normally, NCX couples the efflux of Ca from the cell to sodium (Na) influx, but the direction of ion movement mediated by the exchange is dependent on membrane potential and the extracellular and intracellular concentrations of Na and Ca. The potential at which ion movement switches direction is called the reversal potential. A key concept in cardiac cell Ca regulation is that, because the reversal potential is readily encountered under physiological conditions and can be changed by small changes in intracellular Na concentration, the efflux of Ca from the cell during the cardiac cycle is very dependent on pathways that transport Na and regulate its concentration ^10^. It is important to bear in mind that factors that influence intracellular Na concentration will ultimately affect the intracellular Ca concentration and consequently both active contraction and passive (tonic) force production ^11^, determinants of cardiac output and ventricular filling (see Figure 1).

### The contractile and electrical processes in the failing heart

Heart failure (HF) is the term generally applied to a continual decline in contractile function caused by a variety of conditions but mainly following myocardial infarction. The disease is imposing progressively larger economic and public health burdens because it is becoming more widespread. The prevalence of HF in an unselected population >45 years of age is estimated to be 2.2% and in a population >65 years of age to be 8.8% ^12, 13^. An aging population means a greater number of people will suffer from the disease, but perhaps of more concern is the poor prognosis in this HF population. The median survival following diagnosis with HF is <3 years, and 5-year survival is only 32% in patients with systolic dysfunction and little better (35%) in patients with HF and preserved ejection fraction. The mode of HF-related death can be broadly split into two – “pump failure” and sudden cardiac death (SCD) – with small numbers ascribable to other causes ^14^. SCD is due to rhythm disturbance and there are good reasons to believe that poor cellular Ca regulation and a reduction in repolarizing currents associated with HF not only cause weak contraction but also initiate triggered arrhythmias ^15, 16^. In some circumstances, these events might also contribute to re-entrant forms of arrhythmia ^17, 18^. Arrhythmias can develop (1) as a result of changes in the rate of pacemaker impulses at the sino-atrial node, (2) from electrical variation occurring in areas of the heart not normally associated with impulse generation, or (3) from structural or electrical modifications that alter impulse propagation. The consequence is that extra action potentials are produced that disturb the normal rhythm. Defective cellular Ca regulation can cause extra action potentials to form in two different ways, but both result in spontaneous depolarizations that reach a threshold for Na or Ca channel activation. Spontaneous depolarizations occurring during the repolarization phases of the action potentials are called early afterdepolarizations (EADs) ^19^, while those occurring once the action potential has finished and the cell is electrically quiescent in the diastolic interval are termed delayed afterdepolarizations (DADs) ^16, 20^.

**Figure 2.**
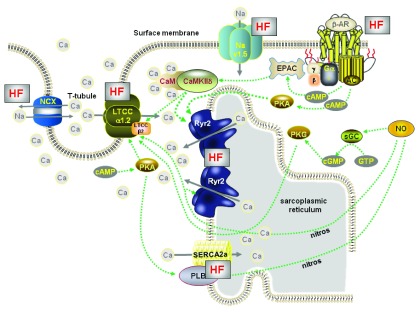
Schematic diagram of the main proteins involved in EC coupling in a ventricular myocyte isolated from the failing heart. The HF label signifies protein function has changed. T-tubule architecture alters resulting in less effective Ca-induced Ca release from the SR, uptake of Ca by SERCA is reduced, late Na current is increased. In addition to other changes in Na regulation this causes an increase in cytosolic [Na] that disturbs the function of NCX. Beta-adrenoceptors are down-regulated in heart failure and CaMKII activity is increased. Local decreases in protein phosphates results in hyperphosphorylation (chronic phosphorylation) of Ryr2 that destabilises the Ca release channel causing opening probability to increase. This results in greater diastolic Ca release from the SR (Ca leak) that, in parallel with reduced SERCA efficiency, lowers SR Ca content.

The reasons for poor contraction and relaxation observed in failing cardiac tissue ^21– 24^ are becoming better understood and involve changes to multiple proteins: some involved in Ca regulation ^25^ and others in the generation of the ionic currents that form the action potential ^26^. Ca uptake into the SR is poorer ^27– 30^, there is more Ca leak from the SR during the diastolic interval ^31, 32^, and there is ineffective release of Ca from the SR ^33– 35^ (see Figure 2).

In HF, the T-tubule network becomes disorganized and disrupted ^36– 38^, so some RyR clusters lose functional contact with their activating Ca channels. These so-called “orphaned” RyR clusters cannot be directly activated by surface Ca channels but can be activated later in the process by Ca released from neighboring clusters. This lag in activating RyRs causes slower and reduced Ca transients and under some circumstances dyssynchronous Ca release, which can alter action potential duration and drive Ca-dependent pro-arrhythmic current formation – EADs and DADs (see Figure 2).

### Ca/calmodulin-dependent protein kinase II

Ca/calmodulin-dependent protein kinases (CaMKs) are intracellular proteins activated by Ca binding to calmodulin. Once activated, they can have a myriad of effects. A widely described effect is the phosphorylation of important proteins involved in EC coupling (Ca channels, RyRs, and PLB). These actions of CaMKII – the predominant isoform in the heart – support normal physiological processes such as Ca-dependent Ca current facilitation ^39^, RyR activation during the cardiac cycle that modulates fractional SR Ca release ^40^, and the frequency-dependent acceleration of relaxation (termed FDAR) ^41^ in which the SR Ca uptake rate increases at faster heart rates. More recently, some effects of CaMKII on Na and potassium (K) channels have been described that have complex consequences on action potential morphology and physiological EC coupling. However, for many years it has also been appreciated that CaMKII-dependent effects may be of pathophysiological importance. Upregulation of CaMKII activity and expression can occur in patients with HF ^42^. Indeed, mice overexpressing the cytoplasmic delta isoform of CaMKII develop dilated cardiomyopathy and die prematurely. Myocytes isolated from the hearts of these mice are hypertrophied and display a failing behavior ^43^.

It is not difficult to appreciate that action potential prolongation and enhanced Ca influx (via phosphorylation of the L-type Ca channel) may precipitate EAD formation and that slower Na channel inactivation (so enhancing Na influx and, in turn, affecting NCX forward and reverse operation during the cardiac cycle) and alterations to RyR gating may promote more frequent and larger spontaneous SR Ca release leading to DAD formation. Given the well-known changes to the beta-adrenergic pathway in chronic HF and its therapeutic axis ^44^, it is perhaps not surprising that some groups are examining if this pathway has connections with the CaMKII pathway. Already we are aware of more indirect linkages with the following:

(1) the cAMP/PKA pathway since, for example, Ca current facilitation and its response to beta-adrenoceptor stimulation were significantly reduced in CaMKIIδ knockout mice ^45^

(2) the parallel Epac (exchange proteins activated by cAMP) pathway since, for example, specific acute Epac activation by 8-CPT reduced Ca transients and SR Ca content while chronic treatment had the opposite effect ^46, 47^; both acute and chronic changes were CaMKII dependent and PKA independent

(3) a pathway involving NO since, for example, NO modulates SR Ca release in response to beta-adrenergic activation ^48^.

In the past, the two pathways have been described and tested quite independently, but now evidence is emerging for complex interactions between beta-adrenergic stimulation and CaMKII – the former activating the latter (see Figure 1).

## Recent advances

### CaMKII

A new study by Grimm *et al.* ^49^ tries to unravel beta-adrenergic effects from those of CaMKII by using CaMKIIδ knockout mice. The acute physiological beta-adrenergic responses are preserved in these mice and they can develop cardiac hypertrophy following trans-aortic constriction (TAC) (though an earlier article showed that they progress to HF more slowly compared with wild-type mice). In this study, cardiac remodeling was induced with chronic beta-adrenoceptor agonist treatment (isoprenaline) and, in the absence of CaMKIIδ, the development of cardiac fibrosis and the progression to HF were inhibited. There was also a reduction in SR Ca leak in the knockout mice that the authors ascribed to CaMKIIδ-mediated phosphorylation of RyR at the serine residue 2814 because mice in which the CaMKII phosphorylation site was genetically inactivated had preserved contractile function and less cardiac dilation following chronic beta-adrenoceptor agonist treatment.

These observations begin to tie in with those of Curran *et al.* ^50^, who used spontaneous Ca waves as an index of arrhythmogenicity. They found that beta-adrenoceptor stimulation leads to increased production of waves that are reduced if nitric oxide synthase (NOS) is inhibited and also showed that NO increases CaMKII-dependent SR Ca leak.

**Figure 3.**
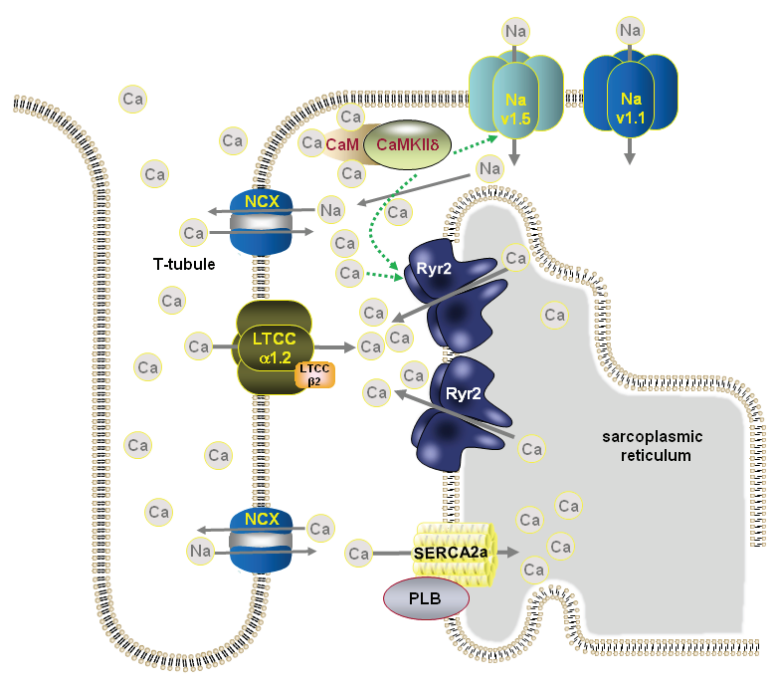
An increase in late Na current mediated by flux through the cardiac (Na _v1.5_) or neuronal (Na _v1.1_) isoforms of the Na channel leads to an increase in sub-sarcolemmal [Na] that reduces the efflux of Ca by NCX during the cardiac cycle. CamKII can phosphorylate Na _v1.5_ which enhances the late Na current.

Interesting work from Fischer *et al.* ^51^ suggests a link between CaMKII and late Na current. When late Na current is increased using anemone toxin II (ATX-II), it leads to increased SR Ca leak that can be prevented by using a CaMKII inhibitor (AIP) or genetically removing the kinase in knockout mice. Surprisingly, the increased SR Ca leak does not appear to alter SR Ca load. An inhibitor of reverse mode NCX function (used at 0.1 μM) also reduced the SR Ca leak mediated by ATX-II. This suggests that CaMKII is activated by Ca, which has entered the cell by reverse mode NCX following an increase in intracellular Na concentration. CaMKII can then subsequently phosphorylate the Na channel (Na _v1.5_) ^52^, which feeds forward, increasing Na influx and so driving more Ca influx (see Figure 3).

Pursuing earlier work by the Hund group, Glynn *et al.* ^53^ provide evidence that the CaMKII-mediated activation of the late Na current occurs via phosphorylation at Ser-571 in Na _v1.5_. They mutated the Ser-571 site in two separate ways producing knock in mice in which the serine was replaced by either glutamate (S571E), so mimicking the phosphorylated protein, or alanine (S571A), so preventing phosphorylation. The S571E mutation increased late Na current but did not change the peak Na current (compared with current recorded from wild-type myocytes), whereas the S571A mutation reduced the late Na current. The results suggested that while Ser-571 phosphorylation can regulate late Na current, it does not significantly modify other channel properties (e.g. steady-state inactivation or recovery from inactivation). An intriguing finding in this work was that the S571A mutation slowed the cardiac remodeling that occurs following pressure overload and the progression towards a failing phenotype.

There is now good evidence for late Na current increasing in HF ^54^ and so these combinations of results provide us with signposts for investigating possible therapeutic interventions aimed at late Na current and CaMKII inhibition in an effort to combat the potentially pro-arrhythmic Ca and Na dysregulation that appears in this pathology. Exactly which Na channels are involved in late Na current flow remains a mystery. Recent work from Mishra *et al.* ^55^ suggests that, apart from Na _v1.5_, there appears to be a substantial contribution (perhaps as much as 60%) of current flow through a neuronal isoform of the Na channel Na _v1.1_. The relationship between neuronal isoforms of the Na channel expressed in the heart and arrhythmogenesis has been explored by Radwanski *et al.* ^56^. They generated a mouse with a calsequestrin mutation (R33Q) that can occur in humans with catecholaminergic polymorphic ventricular tachycardia (CPVT), a disorder characterized by abnormal heart rhythms induced by catecholamines. At the cellular level, the arrhythmias were observed as Ca waves. Nanomolar concentrations of tetrodotoxin (TTX) inhibit neuronal Na channels without significantly blocking the cardiac version (Na _v1.5_). At such concentrations, TTX decreased the frequency of catecholamine-induced Ca waves and increased the number of Ca sparks, which suggested a role for neuronal Na channels in these disturbances of rhythm. Neither CaMKII inhibition with KN-93 in the presence of catecholamine nor the complete omission of catecholamine from the superfusate prevented the neuronal Na channel-mediated aberrant Ca release. The authors also found evidence of co-localization of the neuronal Na channels with RyRs, which could be consistent with their positive feedback role in precipitating diastolic Ca release. It would have been useful if the authors also had looked for co-localization of the neuronal Na channels with NCX (see Figure 3).

### NO involvement

**Figure 4.**
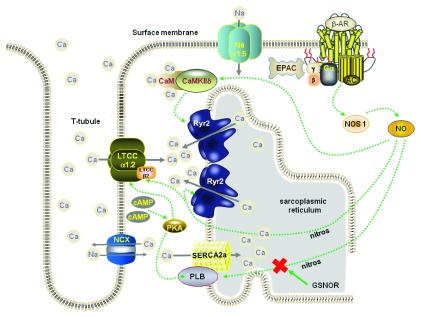
Schematic diagram depicting the activation of nitric oxide synthase 1 (NOS1) following beta-adrenoceptor stimulation. The NO formed can directly activate CaMKII that, in turn, phosphorylates Ryr2 or nitrosylates Ryr2 and PLB. GSNOR (S-nitrosoglutathione reductase) causes denitrosylation.

While phosphorylation is understood to be an important post-translational modification in heart cells affecting a variety of proteins, nitrosylation also brings about changes in protein activity, localization, and stability. Hitherto, its role in regulating signal transduction pathways in the heart has not received much attention, perhaps because the highly reactive NO molecule was thought to lack specificity. However, new work has strongly suggested that more specific nitrosylation or S-nitrosylation (involving a nitroso group and a sulphur atom) of some Ca-handling proteins, in parallel with the phosphorylation routes, is required for transduction of beta-adrenoceptor signaling in cardiac cells. Whilst NO or S-nitrosated proteins (SNOs) lead to protein nitrosylation, denitrosylation can be achieved by S-Nitrosoglutathione reductase (GSNOR). In a study using PLB and GSNOR knockout mice and mice with cardiac-specific GSNOR overexpression ^57^, Irie and colleagues showed that beta-adrenoceptor stimulation induces phosphorylation and S-nitrosylation of PLB. When S-nitrosylation of PLB is inhibited by GSNOR overexpression (or by NO scavenging), then beta-adrenoceptor stimulation does not produce PLB pentamerization and SERCA2a activation even though corresponding phosphorylation of PLB occurs. These important findings suggest that the two parallel processes of phosphorylation and nitrosylation are necessary for complete activation of SERCA2a during beta-adrenoceptor stimulation (see Figure 4).

Nitrosylation also may be important for CaMKII activation *and* inhibition, thereby conferring subtle regulation on the molecule. The action of NO appears to be dependent on whether calmodulin has bound Ca. Evidence provided by Erickson *et al.* ^58^ suggests that CaMKIIδ is activated following nitrosylation of the cys-290 site on the molecule. In contrast, nitrosylation of the cys-273 site inhibits CaMKIIδ activity. The authors suggest that Ca binding to calmodulin produces conformational changes that alter the accessibility of the two binding sites to NO, thereby shifting, in a complex way, the extent to which it can be activated.

### Reactive oxygen species

**Figure 5.**
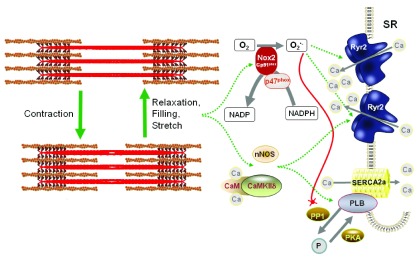
Stretching cardiac myocytes activates NOX2 in discrete locations in the cell to sensitise Ryr2 and increase Ca spark activity. Phospholamban (PLB) phosphorylation that enhances SR Ca uptake occurs by ROS-mediated (O _2_.) inhibition of protein phosphatase-1 activity.

Reactive oxygen species (ROS) is the collective name for reactive molecules and free radicals derived from molecular oxygen. Most ROS are generated as by-products during mitochondrial electron transport. Reduced nicotinamide adenine dinucleotide phosphate (NADPH) oxidase (Nox) proteins generate ROS used in cellular redox signaling and, in the heart, a large number of proteins involved in Ca regulation are modulated by such redox reactions. The main forms of Nox identified in cardiac myocytes are Nox2 and Nox4. Prosser *et al.* ^59^ have shown that stretching cardiac myocytes activates Nox2 in discrete locations in the cell to sensitize RyRs to Ca and increase Ca spark activity. In normal circumstances, the stretch-dependent increase in sensitivity is part of the physiological response to stretch, but in disease states it is conceived as triggering arrhythmogenic Ca waves as a result of the increased frequency of Ca sparks. To examine the effects of increasing Nox2 levels on myocyte function, Zhang *et al.* ^60^ used a transgenic mouse with specific cardiac overexpression of Nox2. They simulated two hypertrophic pathologies *in vivo* by angiotensin II infusions (doses adjusted not to change blood pressure) and, separately, TAC. Angiotensin II treatment and pressure overload caused by TAC both activated Nox2 and induced greater hypertrophy in the transgenic hearts but also improved *in vivo* cardiac function. The cardiac myocytes isolated from mice receiving chronic angiotensin II infusions had increased SR Ca uptake, increased Ca transients and contractile amplitudes, and improved contraction and relaxation. This and other evidence pointed to the enhanced Nox2 activity increasing PLB phosphorylation due to ROS-mediated inhibition of protein phosphatase 1 (see Figure 5).

To explore the physiological and pathophysiological roles of ROS signaling, Limbu *et al.* ^61^ built an *in silico* model of EC coupling and Ca signaling in the heart that took into account stretch-induced ROS signaling. The model was able to simulate the experimentally observed stretch-induced ROS production and bursts of Ca sparks. The model suggested that ROS are produced locally near the RyR complexes, that there is an activation of ROS that corresponds with ventricular filling, and that this leads to enhanced Ca release. If the stretch is prolonged or chronic, the RyR sensitization is not maintained and the increase in Ca spark rate is transient. If the redox environment of the cells is unbalanced and ROS signaling increases, the enhanced SR Ca leak contributes to the formation of a pro-arrhythmic substrate. In this work, much of the emphasis is placed on Nox2 as being an important element in the transduction process. It contrasts with an important article published the previous year in which it was suggested that myocyte stretch activates neuronal NOS (nNOS) and CaMKII independently from Nox2 ^62^.

Jian *et al.* ^62^ found that inhibiting nNOS and CaMKII, but not Nox2, eliminated the stretch-induced burst of Ca sparks in cardiac myocytes with mechanical load controlled by a “cell-in-gel” system. A subtle difference in method, and a potential source of the controversy, is that the cells were stretched in different ways. The nature, amount, and direction of the mechanical load could be important, and this aspect awaits resolution (see Figure 5). However, these articles serve to illustrate the complexity of the signal transduction systems and highlight that mechanical stress influences Ca regulation and cycling. We need to understand how increased afterload experienced in a variety of pathological conditions (for example, elevated blood pressure) leads to cardiac remodeling, and these types of experimental approach allow us to simulate the *in vivo* state and work on identifying the mechanisms involved. The idea of doing patch clamp studies on cells undergoing stretch may not appeal to cell electrophysiologists, but unloaded myocytes clearly yield only part of a very complicated picture.

ROS are suggested also to be central to signaling events between SR Ca and mitochondria in some pathophysiological circumstances. Santulli *et al.* ^63^ provide evidence that the mitochondria in cardiac myocytes gain Ca and produce more ROS following myocardial infarction. They propose that the increased total mitochondrial Ca concentration could result directly from an enhanced SR Ca leak through RyR in diseased myocytes. To test this idea, they used transgenic mice carrying a serine/aspartate mutation of the RyR – S2808D – that increases RyR opening probability to make the SR release channels leaky. They compared the effects of various interventions with other transgenic mice in which serine 2808 has been replaced with alanine (S2808A), making the RyR non-phosphorylatable, which reduces opening probability. Cardiac myocytes isolated from mice with enhanced SR Ca leak had increased mitochondrial Ca concentration and ROS production in turn affecting the function of the mitochondria and their size. Cardiac myocytes from the mouse hearts with reduced leak did not show these mitochondrial changes. Another transgenic mouse model that had greatly reduced IP3 receptor expression did not change the cardiac mitochondrial abnormalities caused by myocardial infarction, suggesting the link between SR Ca leak and mitochondrial dysfunction is the RyR. The final connection was made by crossing MCat mice and the mice with the leaky SR mutation. MCat mice overexpress the human catalase gene in their mitochondria and generate fewer ROS and do not show age-associated reductions in mitochondrial function. Following myocardial infarction, the cross-bred mice had less mitochondrial Ca accumulation than their RyR2-S2808D counterparts and their progression towards HF was markedly slowed.

This work has raised some questions ^64^ that need to be answered to clarify aspects of the mechanisms involved: notably, how is the enhanced Ca leak from the SR translated into increased mitochondrial Ca concentration? However, the work emphasizes evermore-complex interplay of Ca signaling in the heart and the role of Ca stores in those signaling processes. A good review article explaining how mitochondrial calcium influences cardiac metabolism ^65^ was published in 2015 together with an excellent series of articles on Ca and mitochondria in a special issue of the *Journal of Molecular and Cellular Cardiology*.

To understand these pathways fully requires much interdisciplinary collaboration and more initiatives like the National Institutes of Health’s National Heart, Lung, and Blood Institute topic of integrative mitochondrial biology in cardiovascular diseases. The concluding comments of this group of workers have been published ^66^ and illustrates the information that can be gained by harnessing the talents of individuals across a number of disciplines.

### T-tubule disturbances

As mentioned above, in HF the T-tubule network becomes disorganized and disrupted so RyR clusters can lose functional contact with their activating Ca channels. It is not known if the T-tubule disruption causes a reduction in the number of Ca channels, but, if so, this could worsen the disturbed EC coupling. Bryant *et al.* ^67^ examined the changes to Ca current following coronary artery ligation (to induce a HF phenotype) and differentiated the relative densities of Ca current in the T-tubule and surface membranes using osmotic detubulation. They found that whilst there was no change in total Ca current density between cells isolated from hearts of sham-operated animals and those with coronary artery ligation, the cells from ligated hearts showed a redistribution of their Ca channels. In these cells, there were more Ca channels in the surface membrane and fewer in the T-tubules compared with the cells from sham-operated hearts and these changes correlated with increased spatiotemporal inhomogeneity of local Ca release. The work shows another route for disruption of Ca release from the SR in HF.

The effects of T-tubule disruption have been mathematically modeled by the Weiss group ^68^. The group’s results suggest that the underlying mechanism of alternans (a form of cardiac arrhythmia where the amplitude of the Ca transient alternates out of phase in different regions of the same cell) changes during the evolution of HF. In the early stages of the disease, T-tubule disruption plays an important role producing orphaned RyR clusters that, with a steeper SR Ca release-load relationship, lead to cluster-mediated release. These isolated clusters spontaneously release Ca that helps create the alternans. In the later stages of HF when SERCA protein is decreased, cytosolic Ca concentration is increased and this appears to be the more important factor in the genesis of alternans. In support of the findings of Bryant *et al*., the *in silico* modeling provided the opportunity to vary the Ca channel and RyR coupling efficiency by changing the amount of T-tubule disruption. The amount of disruption – and therefore the spatial distribution of the Ca channels – was fundamental to determining the development of alternans.

The very readable review by Wagner *et al.* ^69^ discusses the development of alternans and afterdepolarizations and emphasizes the very close relationship between the control of intracellular levels of Na and Ca ions and the electrical and mechanical properties of the heart. The authors stress that many cell-based arrhythmias result from a breakdown of this close relationship.

Pro-arrhythmic EADs and DADs are often considered in isolation since they have different mechanisms underlying their genesis. A new article from Song *et al.* ^70^ shows some *in silico* predictions and complementary *in vitro* experiments of interactions between these two forms of afterdepolarization. The conclusions are that there is close interplay between them. The occurrence of EADs enhances Ca loading because of more L-type Ca channel openings during the cardiac cycle. The cellular Ca loading promotes DADs. There are also complex feedback mechanisms whereby DADs can suppress EAD occurrences by shortening Ca current inactivation time and there are circumstances when spontaneous Ca release causes EADs through depolarization by NCX that allows reactivation of L-type Ca current. Some of these *in silico* observations remain to be tested *in vitro*, but nevertheless they illustrate the ever-increasing potential interplay between these cellular arrhythmogenic mechanisms.

## Conclusion

In order to keep the article short, I have not included articles from the burgeoning field of work using induced pluripotent stem cell-derived cardiac myocytes. Although this work should prove immensely valuable in investigations of genetic cardiac disease and for pharmacological screening, there are still some hurdles to overcome in projecting the results obtained using such cells with limited maturity to adult cardiac myocytes. Nor have I included a summary of the information delivered in the past year on sarcomeric cardiomyopathies. Central to these are mutations in sarcomeric proteins and although these are important signaling loci in cardiac myocytes and contribute to sarcomeric structure and the regulation of contraction and relaxation, the inclusion of such work would not allow me to concentrate on the more physiological aspects of cardiac contraction.

More detailed examination of, for example, the interactions between the cardiac arrhythmogenic mechanisms or the interplay of various phosphorylation pathways illustrates that nature is extremely complex. The reductionist approach increases our understanding of the mechanism under examination but often causes us to lose sight of how that specific mechanism influences upstream, downstream, or parallel processes. The above small sample of 2015 articles demonstrates the need to maintain also a holistic view because, without some balance, we will struggle to understand nature’s complexities. These days, we use the term “networking” usually in the context of developing social or professional interactions. To get to grips with nature’s cardiac complexities, we will need to develop working arrangements of networks that are responsible for the function of the heart and realize that these networks are not independent.
